# Antiglomerular Basement Membrane Disease Possibly Triggered by Undiagnosed Renal Cell Carcinoma: A Case Report

**DOI:** 10.1016/j.xkme.2023.100709

**Published:** 2023-08-09

**Authors:** Mariell Rivedal, Yngvar Lunde Haaskjold, Hedda Berge, Thomas Knoop

**Affiliations:** 1 Department of Clinical Medicine, University of Bergen, Bergen, Norway; 2 Department of Medicine, Haukeland University Hospital, Bergen, Norway

**Keywords:** Antiglomerular basement membrane disease, renal cell carcinoma, plasmapheresis, immunosuppression, acute kidney injury, rapidly progressive glomerulonephritis, kidney failure, renal failure

## Abstract

Antiglomerular basement membrane (anti-GBM) disease is a rare, small-vessel vasculitis that affects the capillary beds of the kidneys and lungs. Although exceedingly rare, several case reports have described anti-GBM disease with a concurrent cancer diagnosis, suggesting a possible correlation between these 2 conditions. Herein, we describe the first known case to our knowledge of a woman in her early 60s with simultaneous anti-GBM disease and clear cell renal cell carcinoma, in which the tumor was thought to have been the substrate for anti-GBM disease. We believe that renal cell carcinoma may have contributed to the production of anti-GBM autoantibodies and, thus, anti-GBM disease. The concurrence of these 2 conditions complicated the treatment of the patient, who was hemodialysis-dependent at the time of hospital discharge. This report highlights the importance of considering anti-GBM disease as a potential diagnosis in patients with acute kidney failure, and how important it is to identify both clear cell renal cell carcinoma and anti-GBM disease at an early stage to improve outcomes.

## Introduction

Antiglomerular basement membrane (anti-GBM) disease is a rare, small-vessel vasculitis.^1^ Although the association between anti-GBM disease and malignancy is unknown, some case reports describe anti-GBM disease with a concurrent cancer diagnosis,^2^, ^3^, ^4^, ^5^, ^6^ suggesting a possible correlation. However, to the best of our knowledge, no report has described an association between anti-GBM disease and renal cell carcinoma (RCC).

We report the first known case to our knowledge of anti-GBM disease that was possibly triggered by undiagnosed clear cell RCC (ccRCC). The patient was treated with radical nephrectomy, plasmapheresis, corticosteroids, and cyclophosphamide, and she was discharged hemodialysis-dependent.

## Case Report

**Figure 1:**
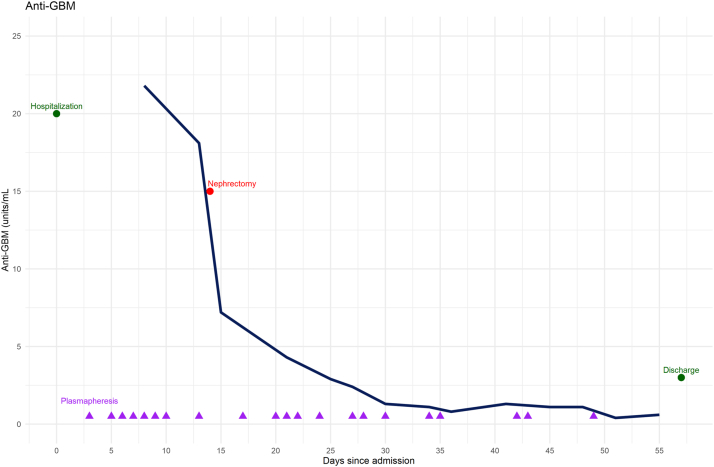
Timeline showing changes in the levels of anti-GBM in addition to important interventions during hospitalization. GBM, glomerular basement membrane

A woman in her early 60s was hospitalized with malaise and anuria (day 1; Fig 1). She was in a deteriorated condition and bedridden, with a body weight of 36 kg (body mass index [BMI], 14.8 kg/m^2^; habitual weight, 45 kg) and a loss of appetite. Her medical history included hypercholesterolemia, hypertension, asthma, and glaucoma, with a positive smoking history (45 pack-years). Because of the acute onset of macroscopic hematuria, nocturnal hyperhidrosis, weight loss, and flank pain, abdominal computed tomography (CT) imaging was performed at another hospital 1 week before admission, revealing a tumor in the right kidney. Simultaneously, serum creatinine levels increased (2 weeks before admission = 0.81 mg/dL; a week before admission = 3.7 mg/dL [measured at the time of the CT imaging]).

**Figure 2:**
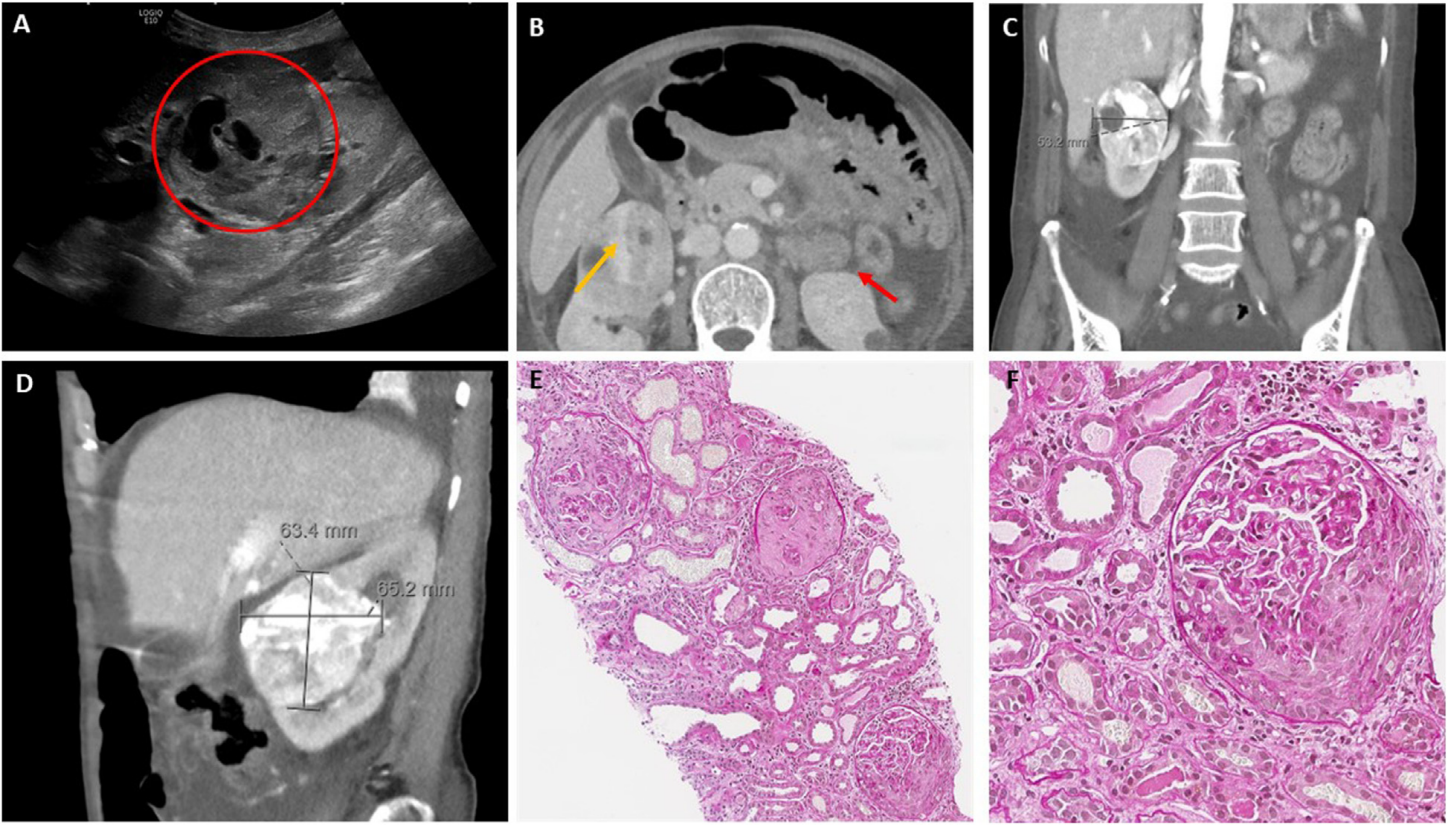
Ultrasonography (A) and computed tomography (CT) (B-D) imaging revealed a tumor in the patient’s right kidney. Fine-needle biopsies were obtained from both tumor and nontumor tissues (E-F). (A) Abdominal ultrasonography at the time of admission showed a tumor (red circle) with peripheral vascularization. No signs of hydronephrosis were observed. Both kidneys were hyperechogenic, swollen, and ∼13 cm long. (B) Axial CT slice (day 2) revealing a tumor in the right kidney (orange arrow) and a simple, hyperdense cyst in the lower pole of the left kidney (red arrow). Moreover, the CT imaging revealed ascites in the pelvis and edema in the subcutaneous adipose tissue near the kidneys and the tumor. There were no signs of hydronephrosis, and pelvic or abdominal CT revealed no lymphadenopathy. (C) Coronal CT imaging (day 2) revealing a mediolateral diameter of 53.2 mm. (D) Sagittal CT imaging (day 2) showing a craniocaudal diameter of 63.4 mm and an anteroposterior diameter of 65.2 mm. (E-F) Fine-needle aspiration biopsy of nontumor tissue (E: original magnification, ×10, F: original magnification, ×20) revealed crescentic lesions in all 10 glomeruli. Significant reactive changes were observed in the tubulointerstitium. Evaluation of possible interstitial fibrosis is challenging because of edema and the presence of inflammatory infiltrates. Moderate wall thickness and hyaline staining were observed in the small arteries. The arterioles showed signs of myocyte vacuolization and endothelial damage. Immunohistochemical staining was negative, except for C5b-9 staining along the capillary walls.

On examination, she was hypertensive (177/98 mm Hg) with a pulse rate of 92 bpm and a respiratory frequency of 14 breaths/min. She had no fever (36.8 °C) at admission; however, she was febrile (38 °C) a week earlier. Pitting edema was observed in the distal lower extremities. Ultrasonography revealed hyperechogenic and swollen kidneys, and a tumor in the right kidney (Fig 2A). Urinary dipstick examination revealed hematuria (+3) and proteinuria (+3). Laboratory tests and arterial blood gas values indicated acute anuric kidney failure (serum creatinine of 13.0 mg/dL [0.5-1.0]) with metabolic acidosis (pH 7.20 [7.36−7.44], HCO_3_^−^ 15 mM [22−26]), hyperkalemia (7.3 mmol/L [3.5−5.0]), and hyponatremia (119 mmol/L [137−145]) (Table 1).

**Table 1:** Main Laboratory and Arterial Blood Gas Results on Admission

Laboratory Tests	Value		Normal Range
Creatinine (mg/dL)	13	↑	0.5-1.0
eGFR (mL/min/1.73 m^2^)	3	↓	
SUN (mg/dL)	110.3	↑	8.7-22.1
Albumin (g/L)	31	↓	39-48
Sodium (mmol/L)	119	↓	137-145
Potassium (mmol/L)	7.3	↑	3.5-5.0
Phosphate (mmol/L)	2.59	↑	0.85-1.50
CRP (mg/L)	137	↑	<5
Hemoglobin (g/dL)	11.7		11.7-15.3
Anti-GBM (units/mL)	>8	↑	<1
Base excess (mmol/L)	−11.8	↓	−3.0 to 3.0
HCO_3_^−^(mmol/L)	15	↓	22-26

*Note:* Conversion factors for units: serum creatinine in mg/dL to mol/L, ×88.4; urea nitrogen in mg/dL to mmol/L, ×0.357.

Abbreviations: eGFR, estimated glomerular filtration rate (calculated using the Chronic Kidney Disease Epidemiology Collaboration formula); SUN, serum urea nitrogen; CRP, C-reactive protein; GBM, glomerular basement membrane; HCO_3_, bicarbonate; ↑, value is higher than the normal range; ↓, value is below the normal range.

Despite the likely malignancy, intravenous methylprednisolone was initiated because of a high suspicion of rapidly progressive glomerulonephritis, and the patient was transferred to the intensive care unit for continuous hemofiltration. Immunological screening revealed an increased anti-GBM level of >8 units/mL (normal range <1 unit/mL), strengthening our suspicion of rapidly progressive glomerulonephritis, specifically anti-GBM disease.

Anti-GBM disease is normally treated with plasmapheresis, cyclophosphamide, and high-dose corticosteroids.^7^ However, because of the observed tumor in the right kidney (Fig 2B-D) and possible malignancy, the patient was not considered a candidate for acute cyclophosphamide treatment. Plasmapheresis was not initiated before a kidney biopsy had been performed, both because hemoptysis was not observed and because a high possibility of permanent loss of kidney function was considered. After administering intravenous methylprednisolone for 3 days, oral prednisone treatment was initiated.

On day 2, a biopsy (Fig 2E and F) was performed on the renal parenchyma and the suspected tumor for histopathological confirmation of the diagnosis. Light microscopy showed 10 glomeruli, all of which contained crescents. The tumor was confirmed to be ccRCC, cT1bN0M0, according to the American Joint Committee on Cancer tumor-node-metastasis staging. The patient had a Leibovich 2018A score of 5,^8^ indicating an intermediate risk of developing metastatic cancer after nephrectomy.

On day 4, plasmapheresis and intermittent hemodialysis were initiated. Four days later, anti-GBM levels peaked at 21.8 units/mL. On day 15, after 8 plasmapheresis sessions, a radical nephrectomy of the right kidney was performed, after thorough consideration (see Discussion). Simultaneously, anti-GBM levels decreased to 7.2 units/mL and continued decreasing until day 36, when the first negative anti-GBM level since admission was measured.

It was concluded that the patient would not require further cancer therapy postnephrectomy because of the radical removal of the tumor and the intermediate risk of metastasis. Thus, we could initiate immunosuppressive therapy, and on day 42, cyclophosphamide was initiated to treat anti-GBM disease. Although cyclophosphamide therapy is associated with an increased risk of malignancy, it has shown efficacy in anti-GBM disease.^9^ Therefore, we considered the benefits to outweigh the risks associated with this therapy.

The patient was discharged after 58 days, without detectable anti-GBM antibodies. Cyclophosphamide was terminated after 3 months, and prednisone was tapered gradually. During hospitalization, the patient underwent 22 plasmapheresis sessions and remained hemodialysis-dependent at discharge.

## Discussion

We have described a rare case of a woman with concurrent anti-GBM disease and ccRCC, in which the tumor was thought to have been the substrate for anti-GBM disease. The patient was treated with radical nephrectomy for ccRCC in addition to plasmapheresis, corticosteroids, and cyclophosphamide for anti-GBM disease. She was hemodialysis-dependent at discharge. To our knowledge, this is the first report describing the concurrence of anti-GBM disease and ccRCC.

Anti-GBM diseases coexisting with malignancies are rare, although they have been described in some case reports.^2^, ^3^, ^4^, ^5^, ^6^ In 1989, McMahon et al^6^ described a case of 2 separate bronchial and pancreatic endocrine tumors in a patient with anti-GBM disease. Later, anti-GBM disease was found in concurrence with bronchial carcinoma,^2^ melanoma,^3^ leukemia,^4^ and small-cell lung cancer.^5^ Although these findings may coincide, the possibility of an association between malignancy and anti-GBM diseases cannot be excluded.

In our patient, the tumor was thought to have triggered the production of anti-GBM autoantibodies. Anti-GBM disease is caused by circulating autoantibodies directed against the noncollagenous domain of the α3 chain of type IV collagen in predominantly glomeruli and alveoli.^1^ The production of these autoantibodies is thought to be triggered by an unknown inciting stimulus,^1^ such as kidney injury and immune dysregulation.^10^ Invasive tumor growth may be an inciting stimulus because it may cause degradation of the matrix structures of the basement membrane, leading to increased collagenase IV expression.^2^^,^^11^ Moreover, there is a significant T-cell component in anti-GBM disease,^10^^,^^12^ with evidence of T-cell dysfunction in cancer.^13^ Thus, we speculate that the growth of the ccRCC tumor, including the immune response to malignancy, may have triggered the production of anti-GBM autoantibodies in this patient, consequently leading to anti-GBM disease. That the anti-GBM antibody levels rapidly decreased after the nephrectomy supports this hypothesis.

Patient and kidney survival rates in patients with concurrent anti-GBM disease and malignancy are unknown. However, patients with anti-GBM disease who were hemodialysis-dependent at presentation and had crescents in 100% of the glomeruli in a kidney biopsy, such as our patient, did not recover their kidney function,^14^^,^^15^ revealing the severity of anti-GBM disease alone. The prognosis is significantly better if the patient is diagnosed earlier. Levy et al^14^ found that patients who presented with a serum creatinine concentration of <5.7 mg/dL had 100% patient survival and 95% kidney survival at 1-year follow-up, compared with 65% patient and 8% kidney survival at 1-year follow-up if the patient presented with hemodialysis-dependent kidney failure; further, a 0% kidney survival was observed in patients showing the presence of crescents in 100% of the glomeruli in kidney biopsy.^14^ Our patient had normal kidney function before disease onset. A week before hospitalization, her serum creatinine increased to 3.7 mg/dL; this measurement was performed before CT imaging at another hospital. However, no intervention was made at that time, and the patient’s serum creatinine levels increased to 13.0 mg/dL at the time of hospitalization. According to the findings of Levy et al,^14^ the patient’s prognosis would have been significantly better if she had been admitted to the hospital and received therapy at the time of the CT imaging, and she would probably not be hemodialysis-dependent after hospital discharge.

Furthermore, the concurrent occurrence of ccRCC and anti-GBM disease complicated the treatment. First, the patient could not immediately receive the normally recommended immunosuppressive treatment for anti-GBM disease because of the malignancy. Normally, anti-GBM disease is treated using plasmapheresis, cyclophosphamide, and high-dose corticosteroids after confirmation of the diagnosis.^7^ However, our patient was not considered a candidate for cyclophosphamide treatment before nephrectomy could be performed. Urologists and nephrologists thoroughly discussed the present case before surgery. Although urologists preferred to delay surgery because of the patient’s unstable condition and to reduce the risk of perioperative complications, nephrologists insisted on immediate surgery because of our suspicion that the tumor functioned as a substrate for her anti-GBM disease. As expected, the patient’s anti-GBM levels decreased significantly postnephrectomy, indicating that this procedure had a positive effect on the anti-GBM disease. Moreover, because of the patient’s low BMI (14.8 kg/m^2^), she received prednisone at a low dose (40 mg daily instead of the usual 60 mg) and cyclophosphamide every other day (instead of every day).

In conclusion, we presented a case of concurrent anti-GBM disease and ccRCC. We speculate that malignancy contributed to the production of anti-GBM autoantibodies and, thus, anti-GBM disease. The concurrence of these 2 conditions complicated the patient’s treatment. Although anti-GBM disease is rare, even more so in concurrence with malignancy, it is important to consider this condition and admit a patient for examination and treatment if serum creatinine increases rapidly. Early identification of ccRCC and anti-GBM diseases is crucial to improving patient and kidney outcomes.

## References

[1] McAdoo S.P., Pusey C.D. (2017). Anti-glomerular basement membrane disease. Clin J Am Soc Nephrol.

[2] Gao C., Xie J., Pan X., Chen X. (2020). Anti-glomerular basement membrane nephritis with bronchial carcinoma: a case report. J Int Med Res.

[3] Kyriazis P., Tiwary A., Freeman J., Landry D., Braden G. (2021). Atypical anti-glomerular basement membrane glomerulonephritis in a patient with metastatic melanoma treated with mitogen-activated protein kinase and immune checkpoint inhibitors: a case report. J Med Case Rep.

[4] Zhang M., Guan N., Zhu P. (2019). Recurrent anti-GBM disease with T-cell large granular lymphocytic leukemia: a case report. Medicine (Baltimore).

[5] Hayashi Y., Katayama Y., Sakuragi M. (2021). Sequential occurrence of microscopic polyangiitis and anti-glomerular basement membrane disease in a patient with small cell lung cancer: a case report. J Med Case Rep.

[6] McMahon R.F., Lawler W., O’Donoghue D.J., Ballardie F.W. (1989). Goodpasture’s syndrome in a patient with two endocrine tumours. Postgrad Med J.

[7] Rovin B.H., Adler S.G., Barratt J. (2021). KDIGO 2021 clinical practice guideline for the management of glomerular diseases. Kidney Int.

[8] Leibovich B.C., Lohse C.M., Cheville J.C. (2018). Predicting oncologic outcomes in renal cell carcinoma after surgery. Eur Urol.

[9] Kant S., Kronbichler A., Geetha D. (2022). Principles of immunosuppression in the management of kidney disease: core curriculum 2022. Am J Kidney Dis.

[10] Pusey CD, Segelmark M. Anti-GBM (Goodpasture) disease: pathogenesis, clinical manifestations, and diagnosis. UpToDate2022.

[11] Fisseler-Eckhoff A., Müller K.M. (1993). [Anti-human collagenase type IV expression in preneoplastic lesions and early squamous cell lung carcinoma]. Verh Dtsch Ges Pathol.

[12] Segelmark M., Hellmark T. (2019). Anti-glomerular basement membrane disease: an update on subgroups, pathogenesis and therapies. Nephrol Dial Transplant.

[13] Thommen D.S., Schumacher T.N. (2018). T cell dysfunction in cancer. Cancer Cell.

[14] Levy J.B., Turner A.N., Rees A.J., Pusey C.D. (2001). Long-term outcome of anti-glomerular basement membrane antibody disease treated with plasma exchange and immunosuppression. Ann Intern Med.

[15] van Daalen E.E., Jennette J.C., McAdoo S.P. (2018). Predicting outcome in patients with anti-GBM glomerulonephritis. Clin J Am Soc Nephrol.

